# Progression of type 1 diabetes is associated with high levels of soluble PD-1 in islet autoantibody-positive children

**DOI:** 10.1007/s00125-023-06075-3

**Published:** 2024-01-12

**Authors:** Sara Bruzzaniti, Erica Piemonte, Dario Bruzzese, Maria Teresa Lepore, Rocky Strollo, Lavinia Izzo, Francesca Di Candia, Adriana Franzese, Maurizio Bifulco, Enza Mozzillo, Johnny Ludvigsson, Giuseppe Matarese, Mario Galgani

**Affiliations:** 1 Laboratorio di Immunologia, Istituto per l’Endocrinologia e l’Oncologia Sperimentale ‘G. Salvatore’, Consiglio Nazionale delle Ricerche, Naples, Italy; 2 Dipartimento di Medicina Molecolare e Biotecnologie Mediche, Università degli Studi di Napoli ‘Federico II’, Naples, Italy; 3 Dipartimento di Sanità Pubblica, Università degli Studi di Napoli ‘Federico II’, Naples, Italy; 4 Dipartimento di Scienze Umane e Promozione della Qualità della Vita, Università Telematica San Raffaele Roma, Rome, Italy; 5 Dipartimento di Scienze Mediche Traslazionali, Università degli Studi di Napoli ‘Federico II’, Naples, Italy; 6 Crown Princess Victoria’s Children’s Hospital and Division of Pediatrics, Department of Biomedical and Clinical Sciences, Linköping University, Linköping, Sweden

**Keywords:** Islet autoantibodies, Prediction of type 1 diabetes, Soluble immune checkpoint molecules, Soluble PD-1, Type 1 diabetes

## Abstract

**Aims/hypothesis:**

Type 1 diabetes is an autoimmune disorder that is characterised by destruction of pancreatic beta cells by autoreactive T lymphocytes. Although islet autoantibodies (AAb) are an indicator of disease progression, specific immune biomarkers that can be used as target molecules to halt development of type 1 diabetes have not been discovered. Soluble immune checkpoint molecules (sICM) play a pivotal role in counteracting excessive lymphocyte responses, but their role in type 1 diabetes is unexplored. In this longitudinal study, we measured sICM levels in AAb-positive (AAb^+^) children to identify molecules related to type 1 diabetes progression.

**Methods:**

We measured the levels of 14 sICM in the sera of AAb^+^ children (*n*=57) compared to those with recent-onset type 1 diabetes (*n*=79) and healthy children (*n*=44), obtained from two cohorts. AAb^+^ children were followed up and divided based on their progression to type 1 diabetes (AAb^P^) or not (AAb^NP^) (if they lost islet autoimmunity and did not develop disease in subsequent years). sICM were also measured in the sample taken at the visit closest to disease onset in AAb^P^ children.

**Results:**

We found that AAb^+^ children had a distinct sICM profile compared with healthy children and those with recent-onset type 1 diabetes. In addition, AAb^+^ children who progressed to type 1 diabetes (AAb^P^) had higher sICM concentrations than non-progressors (AAb^NP^). Further, sICM levels decreased in AAb^P^ children close to disease onset. Application of Cox regression models highlighted that high concentrations of soluble programmed cell death protein 1 (sPD-1) are associated with type 1 diabetes progression (HR 1.71; 95% CI 1.16, 2.51; *p*=0.007).

**Conclusions/interpretation:**

This study reveals an sICM profile that is dysregulated during the preclinical stage of type 1 diabetes, and identifies sPD-1 as a pathophysiologically-relevant molecule that is associated with disease progression, offering a potential target for early interventions in autoimmune diabetes.

**Graphical Abstract:**

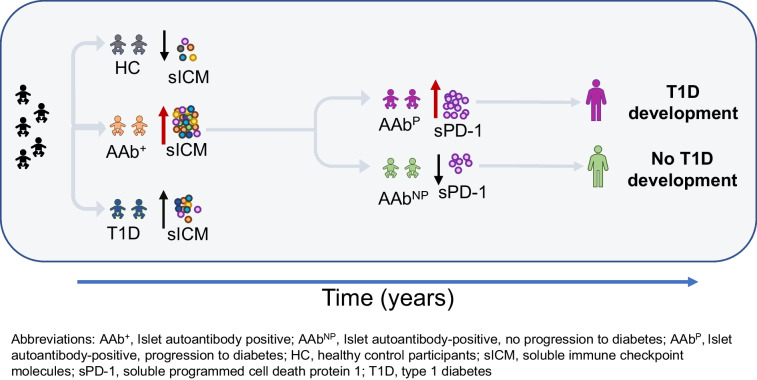

**Supplementary Information:**

The online version contains peer-reviewed but unedited supplementary material available at 10.1007/s00125-023-06075-3.


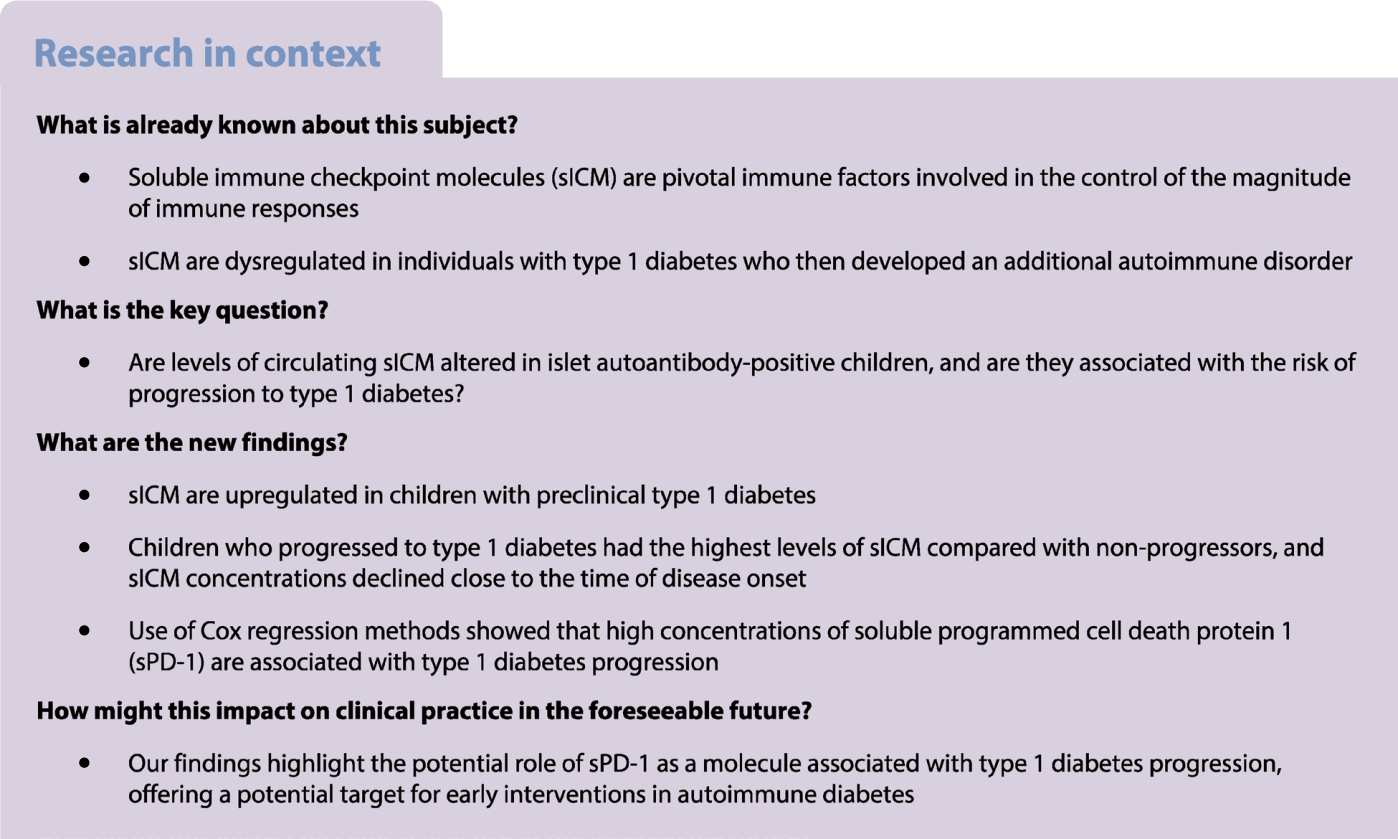


## Introduction

Type 1 diabetes is an autoimmune disorder that is characterised by inflammatory damage to pancreatic beta cells by autoreactive T lymphocytes [1]. Decades of studies have shown that, in genetically susceptible individuals, the disease is a continuum that progresses through distinct identifiable stages before clinical onset [2]. The appearance of plasma autoantibodies against insulin (IAA), a 65 kDa isoform of GAD (GADA), insulinoma-associated antigen-2 (IA-2A) and zinc transporter 8 (ZnT8A) represents an early sign of emerging anti-islet autoimmunity [1]. Indeed, seroconversion towards anti-islet autoantibodies marks an increased risk of developing type 1 diabetes, and the presence of multiple anti-islet autoantibodies is associated with progression to overt disease [2]. However, islet autoantibody titres can fluctuate, and some individuals revert to being autoantibody-negative [3]. Previous studies have suggested that reversion probably occurs in individuals who actually only have a single autoantibody due to assay variability/error [4, 5] or who experienced maternal autoantibody exposure in utero [6]. Although the TEDDY study was the first to show that loss of islet autoimmunity is also linked to other factors such as HLA genotype and age [3], no studies, as far as we are aware, have explored the impact of immune regulatory mechanisms on the basis of this loss of islet autoimmunity.

Immunological self-tolerance is maintained through an intricate network of regulatory pathways that preserve immune homeostasis by preventing autoimmunity. Among them, immune checkpoint molecules (ICM) have been described as pivotal factors for modulating the strength of T cell responses to counteract excessive propagation of immune reactions [7]. Originally identified as membrane-type molecules, soluble forms of ICM (sICM) arise from cleavage of their membrane-bound counterparts or alternative mRNA splicing [8]. Although the role of ICM is well documented, only a limited number of studies have explored the impact of their soluble forms in autoimmune disorders, including type 1 diabetes [9–11]. A growing body of studies have reported dysregulation of sICM as a factor contributing to loss of immunological self-tolerance, due to their ability to act as decoys of their membrane counterpart [8]. Recently, we revealed that levels of sICM, particularly the soluble (s) programmed cell death protein 1 (sPD-1) and sCD137/4-1BB molecules, were significantly higher in children with recent-onset type 1 diabetes, and that these higher levels were associated with the risk of developing an additional autoimmune disorder over time [11].

Based on these observations, we performed a study to investigate potential alterations in sICM levels during the preclinical asymptomatic stages of type 1 diabetes, and their association with type 1 diabetes progression. These findings shed light on the immune mechanisms underlying type 1 diabetes development, and may have implications for potential therapeutic interventions in the future.

## Methods

### Sample cohort

We performed a longitudinal study in 57 islet autoantibody-positive (AAb^+^) children from the All Babies In Southeast Sweden (ABIS) study cohort and the preT1D University of Naples ‘Federico II’ (preT1D-UNINA) study cohort [11–14] (see electronic supplementary material [ESM] Table 1). Genetic predisposition for type 1 diabetes was assessed by genotyping HLA class II regions DQB1, DQA1 and DRB1 at the time of enrolment (ESM Table 2). The ABIS study enrolled babies born in southeast Sweden and the preT1D-UNINA study enrolled Italian babies with the aim of observing development of the disease over time. Samples were collected every 3 years for the ABIS cohort and every year for the preT1D-UNINA cohort until the participants reached puberty; the levels of islet autoantibodies were measured at each visit. To ensure comprehensive data collection, families of both studies were actively encouraged to report data and any potential clinical manifestation directly to research teams through home questionnaires over time, both before and after puberty. The AAb^+^ children included in our study were persistently islet autoantibody-positive on at least two consecutive visits, and analysis of sICM was performed at their first seroconversion visit. Seroconversion was determined by screening for IA-2A, IAA, GADA and ZnT8A by radio-binding assay. Of the 57 AAb^+^ children, 25 progressed to overt type 1 diabetes (AAb^P^) and 32 did not (AAb^NP^), with reversal of persistent islet autoantibodies in subsequent years. Diagnosis of type 1 diabetes was based on ADA criteria [15], and the observed reversion of all islet autoantibodies was defined as two or more consecutive visits with a negative result after seroconversion. All AAb^NP^ children were followed up until they reached puberty, and no instance of another seroconversion or development of type 1 diabetes was reported.

We also included in the study 79 children with recent-onset type 1 diabetes (10 days after disease diagnosis and upon glycaemic stabilisation by treatment with exogenous insulin) and 44 healthy children recruited from the ABIS and preT1D-UNINA cohorts; the healthy children were found to be negative for HLA-associated risk susceptibility for type 1 diabetes and islet autoantibodies, and did not exhibit any autoimmune conditions.

Children in the study were White and were from Sweden and Italy. Gender information was not available for the children enrolled in this study. The sex of participants was determined through self-reporting. Study protocols were approved by the institutional review boards of the ethics committees for the relevant institutions, and informed written consent was obtained from the legal guardians of the children.

### Blood sample processing and serum storage

All blood samples from children recruited in the Swedish and Italian cohorts were processed within 24 h of taking the samples to obtain serum samples that were then aliquoted into cryogenic vials. All serum samples were promptly frozen and stored in a liquid nitrogen tank. For the Swedish sera, the samples were delivered to our Italian laboratory in dry ice, and, upon arrival, were rapidly re-stored in a liquid nitrogen tank, ensuring consistent storage conditions. Thawing of all serum samples was performed on the day of sICM measurement to maintain the integrity of the samples and minimise any potential variations.

### Analysis of sICM

Levels of 14 sICM (B and T lymphocyte attenuator [BTLA], CD27, CD28, CD80, CD137/4-1BB, cytotoxic T lymphocyte antigen-4 [CTLA-4], glucocorticoid-induced TNFR-related [GITR], herpes virus entry mediator [HVEM], indoleamine 2,3-dioxygenase [IDO], lymphocyte activating-3 [LAG-3], PD-1, programmed cell death ligand-1 [PDL-1], programmed cell death ligand-2 [PDL-2] and T cell immunoglobulin and mucin-domain containing-3 [TIM-3]) were measured in sera using a bead-based multianalyte immunoassay (catalogue no. EPX14A-15803-901; ThermoFisher Scientific) according to the manufacturer’s instructions. Sera samples were thawed on the day of sICM measurement, and 25 ml of serum was used. The Luminex xMAP detection system was used to acquire the samples (Luminex 200 System, DiaSorin); xPONENT 3.1 software (Luminex) was used for data acquisition.

### Statistical analysis

Standard descriptive statistics were used to describe the cohort: mean ± SD and range or median with 25^th^ and 75th percentile and range for numerical variables, and absolute frequency with percentage for categorical factors. Accordingly, the ANOVA, the Kruskal–Wallis or the *χ*^2^ tests were used as omnibus tests to assess differences among groups when comparing three independent samples. Post hoc comparisons were based on the *t* test or the Mann–Whitney *U* test for independent samples, without adjustment for multiplicity. To exclude any sample batch effects between samples collected at the different locations, we performed a preliminary analysis in which sICM concentrations between the Italian and Swedish subgroups were compared (ESM Table 3). No difference among the two cohorts was observed (ESM Table 3), allowing us to combine them in a comprehensive analysis. Median regression was applied to evaluate the statistical significance of the differences in sICM concentrations between groups, after adjusting for age and sex. Cox regression models were used to explore the association between each sICM and the risk of developing type 1 diabetes, considering the time of development and censoring those who did not develop the disease based on islet autoantibody reversion. The false discovery rate was controlled using the Benjamini and Hochberg method [16]. Models were adjusted by age, sex, the presence/absence of each islet autoantibody and HLA predisposition; the results are reported as adjusted HRs with the corresponding 95% CI. Correlation analysis was performed using the Pearson test. All statistical analyses were performed in R (version 4.0.3) using the *mfp* package to model fractional polynomials (R Foundation for Statistical Computing, Austria).

## Results

### Experimental study design and population

**Fig. 1:**
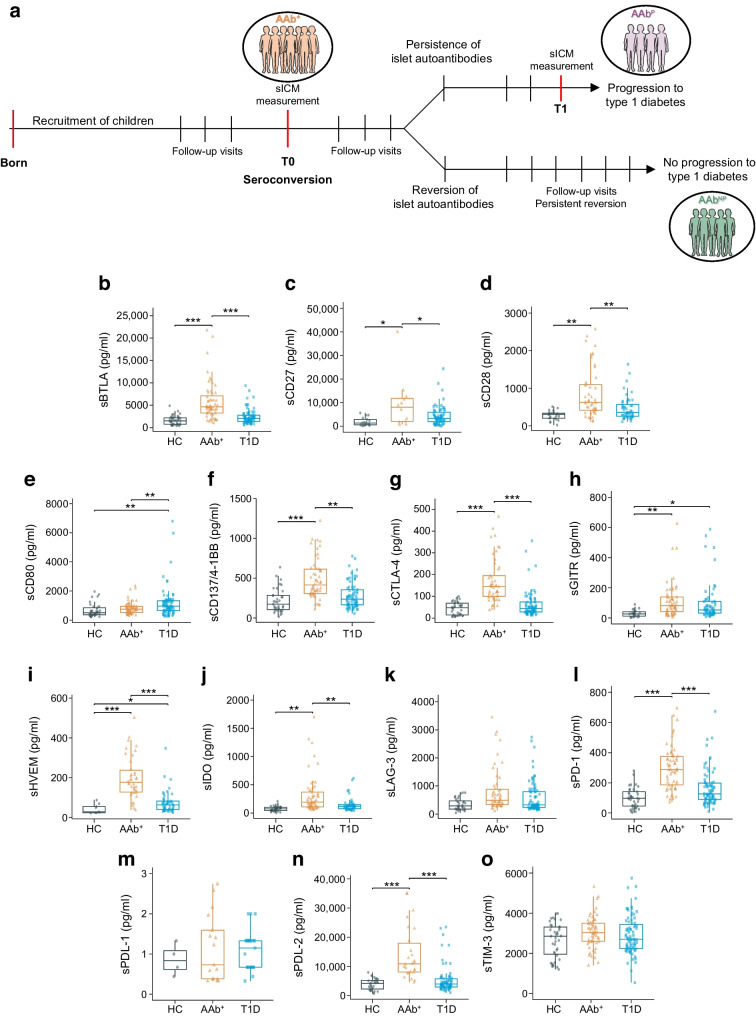
Study design and sICM profile in AAb^+^ children. (**a**) Schematic representation explaining the study design and follow-up visits for AAb^+^ children. The AAb^+^ children included in this study were persistently islet autoantibody-positive on at least two consecutive visits; we measured sICM levels in the sera obtained at their first seroconversion visit (T0). Upon longitudinal follow-up, AAb^+^ children were further categorised based on subsequent progression to type 1 diabetes (AAb^P^) or not (AAb^NP^), based on reversion of all islet autoantibodies in subsequent visits. sICM levels were also measured in AAb^P^ children at the follow-up visit closest to type 1 diabetes onset (T1). (**b**–**o**) Box plots showing the distribution of serum levels (pg/ml) of 14 sICM in healthy children (HC, grey circles), AAb^+^ children (orange triangles) and children with type 1 diabetes (T1D, blue squares). Data are shown as the median (horizontal line in the box) and Q1 and Q3 (borders of the box). Whiskers show the lowest and highest values that are not outliers (i.e. data points below Q1 − 1.5×IQR or above Q3 + 1.5×IQR). Symbols outside the whiskers represent outlier values. The statistical significance of the differences between groups was adjusted by age and sex using median regression (*p<0.05; **p<0.01; ***p<0.001). Figure 1a was created using Servier Medical Art (https://smart.servier.com/). Servier Medical Art by Servier is licensed under a Creative Commons Attribution 3.0 Unported License

In this longitudinal study, we investigated the role of sICM in progression of type 1 diabetes from the asymptomatic stage to overt disease in AAb^+^ at-risk children. To achieve this, we included participants from two distinct cohorts: the ABIS study cohort and the preT1D-UNINA study cohort. The participants included a total of 57 AAb^+^ children with persistent islet autoimmunity verified on two or more consecutive visits and with similar characteristics between the two study cohorts (i.e. age, sex, HLA predisposition). Measurement of sICM was performed on the serum sample obtained at their first seroconversion visit (T0) (Fig. 1a). During the observation period, of the 57 AAb^+^ participants enrolled in this study, 25 progressed to clinical type 1 diabetes (AAb^P^) and 32 AAb^+^ participants experienced reversion of their persistent islet autoantibodies and did not progress to development of the disease (AAb^NP^). Reversion of islet autoantibodies [3] in the AAb^NP^ children was established at two or more consecutive visits after their initial persistent seroconversion. No instance of another seroconversion or development of type 1 diabetes was reported in subsequent years in AAb^NP^ children. In 19 of the 25 AAb^P^ children, sICM measurement was also performed on the blood sample obtained at the visit closest to (immediately preceding) the time of type 1 diabetes onset (T1) (Fig. 1a). As comparison groups, we included 79 children with recent-onset type 1 diabetes and 44 healthy children recruited from the ABIS and preT1D-UNINA cohorts with similar age and sex.

The schematic experimental study design is shown in Fig. 1a, and characteristics of all children included in this study are reported in ESM Table 1 (healthy children, AAb^+^ children and children with type 1 diabetes) and ESM Table 2 (AAb^+^ children who progressed to diabetes [AAb^P^] and those who did not [AAb^NP^]).

### Altered sICM profile in the early preclinical stage of type 1 diabetes

We measured the concentrations of 14 sICM (sBTLA, sCD27, sCD28, sCD80, sCD137/4–1BB, sCTLA-4, sGITR, sHVEM, sIDO, sLAG-3, sPD-1, sPDL-1, sPDL-2 and sTIM-3) in the sera of the 57 AAb^+^ children compared with 79 children with recent-onset type 1 diabetes and 44 healthy children. Multiparametric Luminex analysis revealed that the AAb^+^ children had significantly higher levels of most sICM compared to both children with recent-onset type 1 diabetes and healthy children (Fig. 1b–d,f,g,i,j,l,n). Levels of sGITR were found to be increased only in comparison with healthy children (Fig. 1h). By contrast, sCD80 levels were reduced in AAb^+^ children compared with the type 1 diabetes group (Fig. 1e); no significant differences were observed for sLAG-3, sPDL-1 and sTIM-3 (Fig. 1k,m,o). Additional statistical analysis of a separate comparison between children with type 1 diabetes and healthy children showed that most of the sICM analysed were also increased in the diabetic group compared with the healthy group (ESM Table 4), in line with the findings of our previous study [11].

Together, these results show that a specific sICM signature characterises the asymptomatic preclinical stage of type 1 diabetes.

### Specific sICM are upregulated in the AAb^P^ children and decreased at the visit closest to clinical onset of type 1 diabetes

**Fig. 2:**
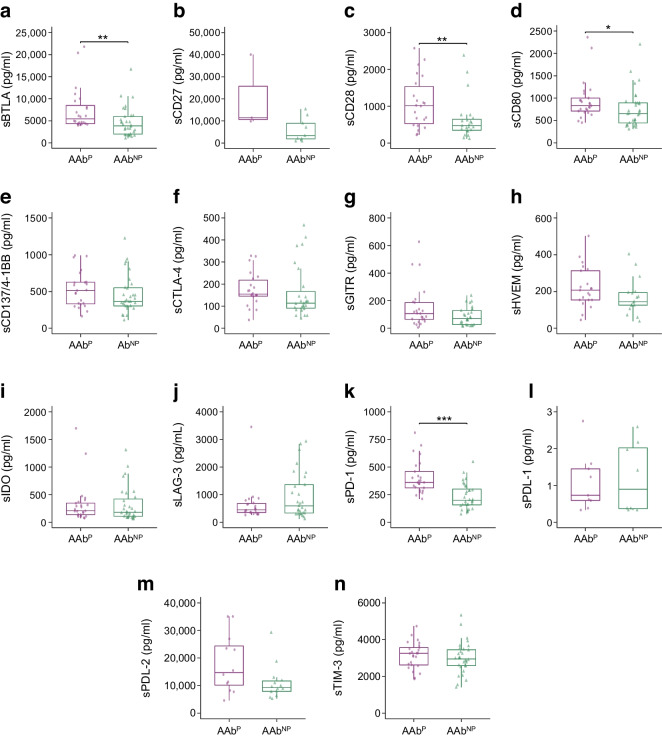
Concentrations of circulating sICM in AAb^+^ children who progressed to type 1 diabetes (AAb^P^) and those who did not (AAb^NP^). (**a**–**n**) Box plots showing the distribution of serum levels (pg/ml) of 14 sICM in AAb^+^ children who developed type 1 diabetes in subsequent years (AAb^P^, purple circles) and those who did not (AAb^NP^, green triangles). Data are shown as the median (horizontal line in the box) and Q1 and Q3 (borders of the box). Whiskers show the lowest and highest values that are not outliers (i.e. data points below Q1 − 1.5×IQR or above Q3 + 1.5×IQR). Symbols outside the whiskers represent outlier values. The statistical significance of the differences between groups was adjusted by age and sex using median regression (*p<0.05, **p<0.01, ***p<0.001)

Next, we divided the 57 AAb^+^ children into two groups based on their future progression to type 1 diabetes (AAb^P^, *n*=25) or not (AAb^NP^, *n*=32) (participants in whom reversion of persistent islet autoantibodies was observed and no seroconversion or clinical type 1 diabetes onset occurred in subsequent years). Our results revealed that AAb^P^ children had significantly higher levels of sBTLA, sCD28, sCD80 and sPD-1 molecules compared with AAb^NP^ children (Fig. 2a,c,d,k).

**Fig. 3:**
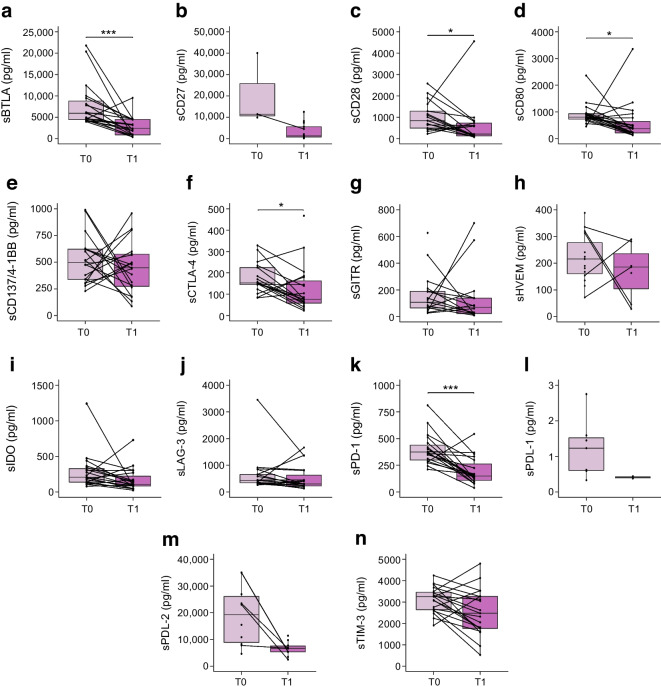
Concentrations of circulating sICM in AAb^+^ children at seroconversion (T0) and time closest to type 1 diabetes diagnosis (T1). (**a**–**n**) Box plots showing the distribution of serum levels (pg/ml) of 14 sICM in 19 AAb^P^ children at their first seroconversion visit (T0, light purple) compared with the levels at the visit closest to type 1 diabetes onset (T1, dark purple). Data are shown as the median (horizontal line in the box) and Q1 and Q3 (borders of the box). Whiskers show the lowest and highest values that are not outliers (i.e. data points below Q1 − 1.5×IQR or above Q3 + 1.5×IQR). Symbols outside the whiskers represent outlier values. The statistical significance of the differences between groups was adjusted by age and sex using median regression (*p<0.05, ***p<0.001)

In 19 of the 25 AAb^P^ children, we analysed sICM levels in the serum sample taken at the visit closest to the clinical diagnosis (T1). We found that levels of sBTLA, sCD28, sCD80, sCTLA-4 and sPD-1 were significantly reduced at T1 compared with baseline (T0) levels (Fig. 3a,c,d,f,k). The overall decrease in sICM concentrations observed in AAb^P^ children at T1 reached levels comparable to those in the recent-onset type 1 diabetes group (ESM Table 5).

These findings suggest that the sICM profile is dysregulated in the early preclinical stages of type 1 diabetes, and, in particular, that higher concentrations of these regulatory soluble immune molecules characterise a specific window that corresponds to the initial phase of an altered immune response.

### High levels of sPD-1 in the early preclinical stage of type 1 diabetes are associated with an increased risk of developing the disease over time

**Fig. 4:**
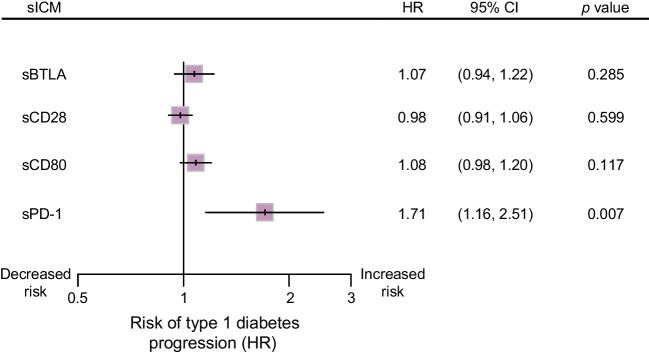
Association of high levels of sPD-1 in AAb^+^ children with increased risk of developing type 1 diabetes over time. Forest plot showing HRs with the corresponding 95% CI for the risk of developing type 1 diabetes, estimated in children who were positive for each sICM for which differences were statistically significant in the comparison between the AAb^P^ and AAb^NP^ groups. An HR >1 indicates that the higher the value for the sICM, the higher the risk of developing type 1 diabetes. On this basis, sPD-1 has been identified as a potential prognostic biomarker of diabetes progression. The statistical significance was adjusted by age, sex, the presence/absence of islet autoantibodies and HLA predisposition for type 1 diabetes

To gain insights into the relationship between sICM and type 1 diabetes progression in the early preclinical stage of the disease, Cox regression models were applied, focusing on the four sICM that exhibited significant differences between AAb^P^ and AAb^NP^ children at their first seroconversion visit (Fig. 2). Models were adjusted by age, sex, the presence or absence of a single or multiple islet autoantibodies and HLA genotype for type 1 diabetes. The results are reported as adjusted HRs with corresponding 95% CIs. These models revealed that among the four sICM that exhibited significant differences, only the high levels of sPD-1 were associated with the increased risk of developing type 1 diabetes over time in AAb^+^ children (HR 1.71; 95% C.I. 1.16, 2.51) (Fig. 4). The significant association between sPD-1 and the risk of type 1 diabetes progression remained significant after controlling for the false discovery rate using the Benjamini and Hochberg method.

Furthermore, we explored the relationship between sPD-1 levels and the time at which seroconversion occurs in AAb^+^ children. Interestingly, our findings revealed that, specifically in AAb^+^ children, sPD-1 levels are inversely correlated with age at seroconversion (T0) (ESM Fig. 1a). The highest increase in sPD-1 concentrations was observed in AAb^+^ children who seroconverted before the age of 10 (ESM Fig. 1b,c), particularly those who progressed to the disease in subsequent years (ESM Fig. 1d).

These data suggest sPD-1 is a previously unrecognised immune factor determining either the reversion of islet autoimmunity or the progression of overt disease. Also, these data potentially reflect a more aggressive autoimmunity in children who undergo seroconversion at a younger age.

## Discussion

In this paper, we report that preclinical type 1 diabetes is characterised by altered concentrations of sICM, particularly high levels of sPD-1, which are associated with disease progression in AAb^+^ children. Additionally, we observed a decrease in sICM concentrations in AAb^P^ children at the visit closest to the disease onset, reaching levels comparable to those observed in type 1 diabetes children.

There is a common consensus that, in the natural history of type 1 diabetes, pancreatic beta cell damage begins months to years before disease onset, as a continuum that progresses from asymptomatic stages to clinical development [2]. The presence of islet autoantibodies is an important indicator of ongoing autoimmune activity; however, disease progression is quite heterogeneous among AAb^+^ individuals, and as yet unpredictable. Indeed, while an appreciable proportion of AAb^+^ individuals develop type 1 diabetes later, reversion of persistent islet autoantibodies may occur in others [3, 17]. Previous studies have explained this phenomenon as secondary to specific HLA genotype, age and metabolic disturbances, which they described as associating factors that are responsible for disease progression or not [3, 17]. However, an altered immune response of autoreactive lymphocytes is considered to be the real player involved in the damage of beta cells and subsequent progression to autoimmune diabetes [18]. Therefore, we may hypothesise that other factors are involved in the pathophysiological events that define disease progression. It has been recognised that several immune regulatory pathways work together in an attempt to ensure tissue integrity and pancreatic tissue homeostasis. In this scenario, sICM may represent poorly explored immune mediators for the control of immune responses during diabetes development. Hence, the ‘sICM storm’ that we observed at the preclinical stages of type 1 diabetes, specifically the high concentrations of sPD-1, may reflect a dysregulated immune activation that may lead to loss of immunological self-tolerance. In contrast to the membrane form, sPD-1 acts as a decoy molecule that is able to interfere with the delivery of inhibitory signalling by its surface counterpart on immune cells [8]. The negative impact resulting from increased concentrations of this molecule has been observed in other autoimmune conditions, linked to disease severity [19, 20]. In a previous study, we also reported that high concentrations of sPD-1 are associated with the development of additional autoimmune disorders in children with type 1 diabetes [11]; however, to our knowledge, no studies have explored the contribution of sPD-1 to the progression of type 1 diabetes development. We hypothesise that the high concentrations of this molecule favour the development of clinical type 1 diabetes by limiting the delivery of inhibitory signals to hyperactivated lymphocytes, and thus our study starts to explain, from an immunological perspective, why some individuals progress to clinical type 1 diabetes while others lose their islet antibody positivity over time.

In this context, the present study first highlights the role of sPD-1 as a ‘predictor’ of overt type 1 diabetes, dictating disease progression at a pathological level. The high sPD-1 levels decrease closer to disease onset, highlighting a specific ‘window’ of immune dysregulation in AAb^+^ children.

One limitation of this study is the relatively small cohort of AAb^+^ children analysed, which may restrict the statistical power necessary to establish a robust association between sPD-1 levels and progression to type 1 diabetes. Also, as our results did not provide a specific predictive value for sPD-1 levels in this context, it is essential to perform further analyses using larger cohorts to obtain a precise risk prediction value. Furthermore, future mechanistic studies could enhance the understanding of the role of sPD-1 as a pivotal factor in the early immune dysregulation stage during preclinical type 1 diabetes before disease progression. In-depth investigation of the underlying mechanisms by which sPD-1 influences immune dysregulation would provide valuable insights into the pathogenesis of type 1 diabetes and potentially open new avenues for therapeutic interventions.

Overall, this study enhances our understanding of the immunological events characterising the preclinical stage of type 1 diabetes, highlighting sPD-1 as a relevant pathophysiological molecule involved in the mechanisms at the root of type 1 diabetes development and progression.

### Supplementary Information

Below is the link to the electronic supplementary material.

Supplementary file1 (PDF 321 KB)

## Data Availability

Any additional information required to reanalyse the data reported in this paper is available from Mario Galgani upon request.

## Abbreviations

AAb^+^: Islet autoantibody-positive
AAb^NP^: Islet autoantibody-positive, no progression to diabetes
AAb^P^: Islet autoantibody-positive, progression to diabetes
BTLA: B and T lymphocyte attenuator
CTLA-4: Cytotoxic T lymphocyte antigen-4
GITR: Glucocorticoid-induced TNF receptor-related
HVEM: Herpes virus entry mediator
IA-2A: Insulinoma-associated antigen-2 autoantibodies
IDO: Indoleamine 2,3-dioxygenase
LAG-3: Lymphocyte activating-3
PD-1: Programmed cell death protein 1
PDL-1: Programmed cell death ligand-1
PDL-2: Programmed cell death ligand-2
s: Soluble(protein)
sICM: Soluble immune checkpoint molecules
TIM-3: T cell immunoglobulin and mucin-domain containing-3
