# Supplementary material for: Progression of type 1 diabetes is associated with high levels of soluble PD-1 in islet autoantibody-positive children
Source: Diabetologia. 2024 Jan 12;67(4):714–23. doi: 10.1007/s00125-023-06075-3 (PMC10904438; doi:10.1007/s00125-023-06075-3)
Supplement: Supplementary file 1 — Supplementary file1 (PDF 321 KB) [file 125_2023_6075_MOESM1_ESM.pdf]

Characteristics	Healthy children	AAb^+ children	T1D children	*p* value
Number of subjects	44	57	79	NA
Sex				0.019
Female	17 (38.6%)	16 (28.1%)	41(51.9%)	
Male	27 (61.4%)	41 (71.9%)	38 (48.1%)	
Age at sICM measurement (years)	9.2 (2.4 to 18)	7.9 (3.1 to 17.9)	9.6 (2 to 17.4)	0.094
Recruitment				
ABIS	16	44	29	NA
preT1D-UNINA	28	13	50	NA

ESM Table 1.

Characteristics of children enroled in the study at the time of sICM measurement. Data are reported using median (range) in the case of numerical variables and absolute frequencies and percentages for categorical factors. Kruskall-Wallis ANOVA and Chi -square test were used as *omnibus* tests.

Characteristics	AAb^{P} children	AAb^{NP} children	*p* value
Number of subjects	25	32	NA
Sex			0.759
Female	6 (24%)	10 (31.2%)	
Male	19 (76%)	22 (68.8%)	
Age at sICM measurement (years)	5.7 (5 to 8.9)	8.1 (3.1 to 17.9)	<0.001
Recruitment			
ABIS	20	24	NA
preT1D-UNINA	5	8	NA
Age at T1D diagnosis or AAb^{+} reversion	12.3 ± 3.6 (6 to 21)	14.3 ± 2.9 (6 to 18)	0.02
Interval time T0 and T1 (years)	5.5 (min:2.5, max:7.9)	NA	NA
HLA typing			
DR3/4	3	6	NA
DR4/4	4	5	NA
DR3/3	1	1	NA
DR4/X	10	8	NA
DR3/X	4	9	NA
DR4/15	0	1	NA
DR3/15	3	0	NA
DRX/15	0	1	NA
DR15/15	0	1	NA
HLA risk			
Strongly increased risk[a]	3	3	NA
Moderately increased risk[b]	15	11	NA
Slightly increased risk[c]	4	12	NA
Neutral risk[d]	3	3	NA
Decreased risk[e]	0	3	NA
Number of autoantibodies			
1	3	20	NA
2	15	10	NA
3	4	2	NA
4	3	0	NA
Islet autoantibody positivity (n subjects)			
GADA	(22)	(25)	NA
IA-2A	(20)	(10)	NA
IAA	(12)	(11)	NA
Znt8A	(3)	(0)	NA
Islet autoantibody values			
GADA	3870 ± 8436 (5 to 35800)	3689 ± 15534 (0 to 87900)	0.001
IA-2A	2772 ± 10253 (2.7 to 51550)	116.4 ± 437.5 (1 to 2460)	<0.001
IAA	10.25 ± 20.08 (0 to 94.8)	5.657 ± 7.875 (0 to 22.5)	0.3984

ESM Table 2.

Characteristics of AAb^+ children at the time of sICM measurement divided in children who developed type 1 diabetes in subsequent years (AAb^P) and those who did not (AAb^{NP}). Data are reported using mean ± standard deviation (SD) with range or median with range in case of numerical variables and absolute frequencies and percentages for categorical factors. Accordingly, differences between groups were assessed through *t*-test for unpaired samples or Chi-square test. [a]Heterozygosity for the two risk-associated haplotypes DR4-DQ8 and DR3-DQ2 (DR3/4). [b]Homozygosity for the two risk-associated haplotypes DR4-DQ8 and DR3-DQ2 (DR4/4 and DR3/3) or risk-associated haplotype DR4-DQ8 combined with a neutral haplotype (DR4/X). [c]Risk-associated haplotype DR3-DQ2 combined with a neutral haplotype DR15-DQ6 (DR3/X). [d]Homozygosity for neutral haplotypes (DRX/X) or risk-associated haplotypes DR4-DQ8 and DR3-DQ2 combined with the protective haplotype DR15-DQ6 (DR4/15 and DR3/15). [e]Neutral haplotype combined with protective haplotype (DRX/15). NA, not applicable.

sICM	Healthy children		AAb⁺ children		T1D children	
	ABIS Cohort	*PreT1D-UNINA Cohort*	*ABIS Cohort*	*PreT1D-UNINA Cohort*	*ABIS Cohort*	*PreT1D-UNINA Cohort*
sBTLA	1749 [859;2316]<br>(418 to 4882)	1619 [772;2390]<br>(482 to 3696)	4608 [3292;7791]<br>(1084 to 21784)	4776 [3092;6416]<br>(2049 to 12460)	1855 [1215;2654]<br>(750 to 8277)	2240 [1474;2905]<br>(662 to 9388)
sCD27	1473 [433;4221]<br>(189 to 5632)	959 [540;2877]<br>(355 to 4735)	9253 [1117;14695]<br>(828 to 40062)	6151 [2432;9633]<br>(2130 to 9856)	2995 [1241;5533]<br>(430 to 24345)	3701 [2204;6312]<br>(129 to 18143)
sCD28	281 [163;348]<br>(87 to 471)	300 [202 ; 349]<br>(19 to 508)	655 [434;1063]<br>(116 to 2577)	499 [227;1554]<br>(138 to 2266)	343 [246;663]<br>(109 to 1003)	356 [243;537]<br>(181 to 1638)
sCD80	442 [34 ;851]<br>(222 to 1962)	555 [380;880]<br>(218 to 1626)	774 [549;939]<br>(309 to 2362)	740 [494;1005]<br>(369 to 2121)	958 [686;1427]<br>(327 to 3985)	950 [514;1274]<br>(250 to 6797)
sCD137/<br>4-1BB	161 [102;365]<br>(43 to 529)	200 [98;264]<br>(16 to 640)	405 [318;610]<br>(110 to 1223)	451 [225;717]<br>(164 to 965)	175 [142;309]<br>(107 to 748)	255 [186;389]<br>(57 to 777)
sCTLA4	46 [13;72]<br>(6 to 100)	48 [13;60]<br>(8 to 92)	123 [97;229]<br>(38 to 467)	146 [98;184]<br>(57 to 374)	30 [21;56]<br>(14 to 308)	52 [34;88]<br>(14 to 355)
sGITR	26 [9;43]<br>(2 to 90)	31 [11;45]<br>(5 to 65)	82 [47;147]<br>(17 to 627)	82 [20;123]<br>(6 to 461)	45 [25;133]<br>(11 to 589)	53 [32;107]<br>(8 to 475)
sHVEM	28 [23;78]<br>(21 to 87)	36 [23;54]<br>(23 to 55)	177 [128;237]<br>(45 to 404)	183 [112;339]<br>(38 to 502)	54 [35;81]<br>(29 to 206)	71 [44;93]<br>(24 to 347)
sIDO	70 [37;102]<br>(12 to 208)	79 [45;106]<br>(3 to 162)	198 [126;345]<br>(57 to 1310)	172 [83;480]<br>(60 to 1702)	118 [69;169]<br>(42 to 403)	123 [83;151]<br>(42 to 617)
sLAG-3	286 [144;449]<br>(41 to 638)	289 [180;560]<br>(102 to 760)	454 [340;861]<br>(195 to 3455)	530 [306;1646]<br>(119 to 2931)	319 [271;847]<br>(138 to 2606)	331 [204;790]<br>(164 to 2739)
sPD-1	98 [45;152]<br>(4 to 254)	97 [45;145]<br>(8 to 280)	290 [187;382]<br>(72 to 811)	287 [137;362]<br>(97 to 619)	125 [102;201]<br>(11 to 488)	128 [73;194]<br>(25 to 675)
sPDL-1	0.7 [0.4;1]<br>(0.4 to 1)	1 [0.7;1.3]<br>(0.7 to 1.3)	0.6 [0.4;1.7]<br>(0.3 to 2.8)	1.2 [0.4;2]<br>(0.3 to 2.2)	0.7 [0.7;1.3]<br>(0.7 to 1.3)	1.2 [0.7;1.3]<br>(0.3 to 2)
sPDL-2	4078 [1562;5501]<br>(705 to 6732)	4273 [2292;5411]<br>(956 to 7947)	11447 [8750;24360]<br>(4616 to 35057)	9456 [7769;12566]<br>(5228 to 23033)	4010 [3150;5483]<br>(2001 to 23497)	3907 [2763;6377]<br>(1011 to 23033)
sTIM3	2852 [2281;3317]<br>(1311 to 3983)	2853 [1537;3382]<br>(1204 to 3978)	3023 [2616;3483]<br>(1404 to 5328)	3135 [2382;3832]<br>(1542 to 4828)	2683 [2235;3350]<br>(1545 to 5262)	2788 [2245;3515]<br>(545 to 5746)

ESM Table 3.

Levels of sICM measured in healthy, AAb$^+$ and recent-onset type 1 diabetes (T1D) children subdivided for the ABIS and preT1D-UNINA cohorts, respectively. Data are reported using median [Interquartile range] (min to max).

sICM	Healthy children (n=44)	T1D children (n=79)	*p* value
sBTLA	1539 [750 ; 2282] (418 to 4882)	2049 [1372 ; 2822] (662 to 9388)	0,010
sCD27	1371 [540 ; 3162] (189 to 5632)	3329 [1833 ; 6251] (129 to 24345)	0,004
sCD28	300 [202 ; 339] (19 to 508)	352 [246 ; 604] (109 to 1638)	0,008
sCD80	505 [360 ; 875] (218 to 1962)	954 [644 ; 1336] (250 to 6797)	<0.001
sCD137/4-1BB	175 [98 ; 284] (16 to 640)	237 [164 ; 358] (57 to 777)	0,027
sCTLA4	48 [13 ; 69] (6 to 100)	44 [28 ; 74] (14 to 355)	0,165
sGITR	26 [11 ; 45] (2 to 90)	53 [31 ; 110] (8 to 589)	<0.001
sHVEM	28 [23 ; 62] (21 to 87)	63 [40 ; 86] (24 to 347)	0,016
sIDO	73 [45 ; 103] (3 to 208)	120 [75 ; 153] (42 to 617)	<0.001
sLAG-3	288 [173 ; 504] (41 to 760)	323 [229 ; 806] (138 to 2739)	0,033
sPD-1	98 [45 ; 146] (4 to 280)	127 [88 ; 201] (11 to 675)	0,003
sPDL-1	0.84 [0.5 ; 1.25] (0.44 to 1.33)	1.15 [0.67 ; 1.33] (0.33 to 2)	0,409
sPDL-2	4175 [2149; 5325] (705 to 7947)	3951 [2922 ; 5910] (1011 to 23497)	0,241
sTIM3	2853 [1900 ; 3327] (1204 to 3983)	2704 [2242 ; 3490] (545 to 5746)	0,315

ESM Table 4.

Levels of sICM measured in healthy and recent-onset type 1 diabetes (T1D) children. Data are reported using median [Interquartile range] (min to max). $p<0.05$ denoting statistical significance by Mann-Whitney U test.

AAb^P children (T0)	sICM	AAb^P children (T1)	Healthy children	T1D children	*p* value
5445 [4325 ; 9146] (3963 to 21784)	sBTLA	2380 [793 ; 4544] (378 to 9537)	1539 [750 ; 2282] (418 to 4882)	2049 [1372 ; 2822] (662 to 9388)	NS
11417 [9856 ; 40062] (9856 to 40062)	sCD27	1283 [613 ; 6453] (188 to 12530)	1371 [540 ; 3162] (189 to 5632)	3329 [1833 ; 6251] (129 to 24345)	NS
1013 [485 ; 1670] (222 to 2577)	sCD28	212 [110 ; 765] (68 to 4558)	300 [202 ; 339] (19 to 508)	352 [246 ; 604] (109 to 1638)	NS
831 [698 ; 1052] (453 to 2362)	sCD80	375 [212 ; 779] (128 to 3356)	505 [360 ; 875] (218 to 1962)	954 [644 ; 1336] (250 to 6797)	***Ab^P *vs* T1D
513 [325 ; 629] (164 to 990)	sCD137/4-1BB	448 [264 ; 581] (86 to 958)	175 [98 ; 284] (16 to 640)	237 [164 ; 358] (57 to 777)	**Ab^P *vs* T1D **Ab^P *vs* HC
155 [139 ; 227] (38 to 328)	sCTLA4	76 [55 ; 180] (23 to 467)	48 [13 ; 69] (6 to 100)	44 [28 ; 74] (14 to 355)	NS
107 [58 ; 189] (6 to 627)	sGITR	70 [22 ; 152] (9 to 700)	26 [11 ; 45] (2 to 90)	53 [31 ; 110] (8 to 589)	NS
207 [154 ; 317] (45 to 502)	sHVEM	186 [45 ; 281] (28 to 289)	28 [23 ; 62] (21 to 87)	63 [40 ; 86] (24 to 347)	*Ab^P *vs* T1D *Ab^P *vs* HC
206 [131 ; 355] (72 to 1702)	sIDO	108 [82 ; 270] (24 to 729)	73 [45 ; 103] (3 to 208)	120 [75 ; 153] (42 to 617)	NS
451 [345 ; 698] (259 to 3455)	sLAG-3	301 [214 ; 805] (127 to 1663)	288 [173 ; 504] (41 to 760)	323 [229 ; 806] (138 to 2739)	NS
361 [299 ; 486] (211 to 811)	sPD-1	149 [98 ; 299] (38 to 543)	98 [45 ; 146] (4 to 280)	127 [88 ; 201] (11 to 675)	NS
0.73 [0.48 ; 1.52] (0.33 to 2.75)	sPDL-1	0.41 [0.38 ; 0.44] (0.38 to 0.44)	0.84 [0.5 ; 1.25] (0.44 to 1.33)	1.15 [0.67 ; 1.33] (0.33 to 2)	NS
14734 [8873 ; 26087] (4616 to 35057)	sPDL-2	6650 [4880 ; 8252] (2451 to 11403)	4175 [2149 ; 5325] (705 to 7947)	3951 [2922 ; 5910] (1011 to 23497)	*Ab^P *vs* T1D
3253 [2603 ; 3624] (1861 to 4735)	sTIM3	2476 [1766 ; 3271] (524 to 4802)	2853 [1900 ; 3327] (1204 to 3983)	2704 [2242 ; 3490] (545 to 5746)	NS

ESM Table 5.

Left, levels of sICM measured in AAb$^+$ children who developed type 1 diabetes (AAb^P) at the time of their first-seroconversion (T0); right, levels of sICM measured in AAb^P closest to disease diagnosis (T1) compared with healthy and recent-onset type 1 diabetes (T1D) children. Data are reported using median [Interquartile range] (min to max), Kruskall Wallis ANOVA was utilized as *omnibus* test (**p*<0.05; ***p*<0.01; ****p*<0.001).

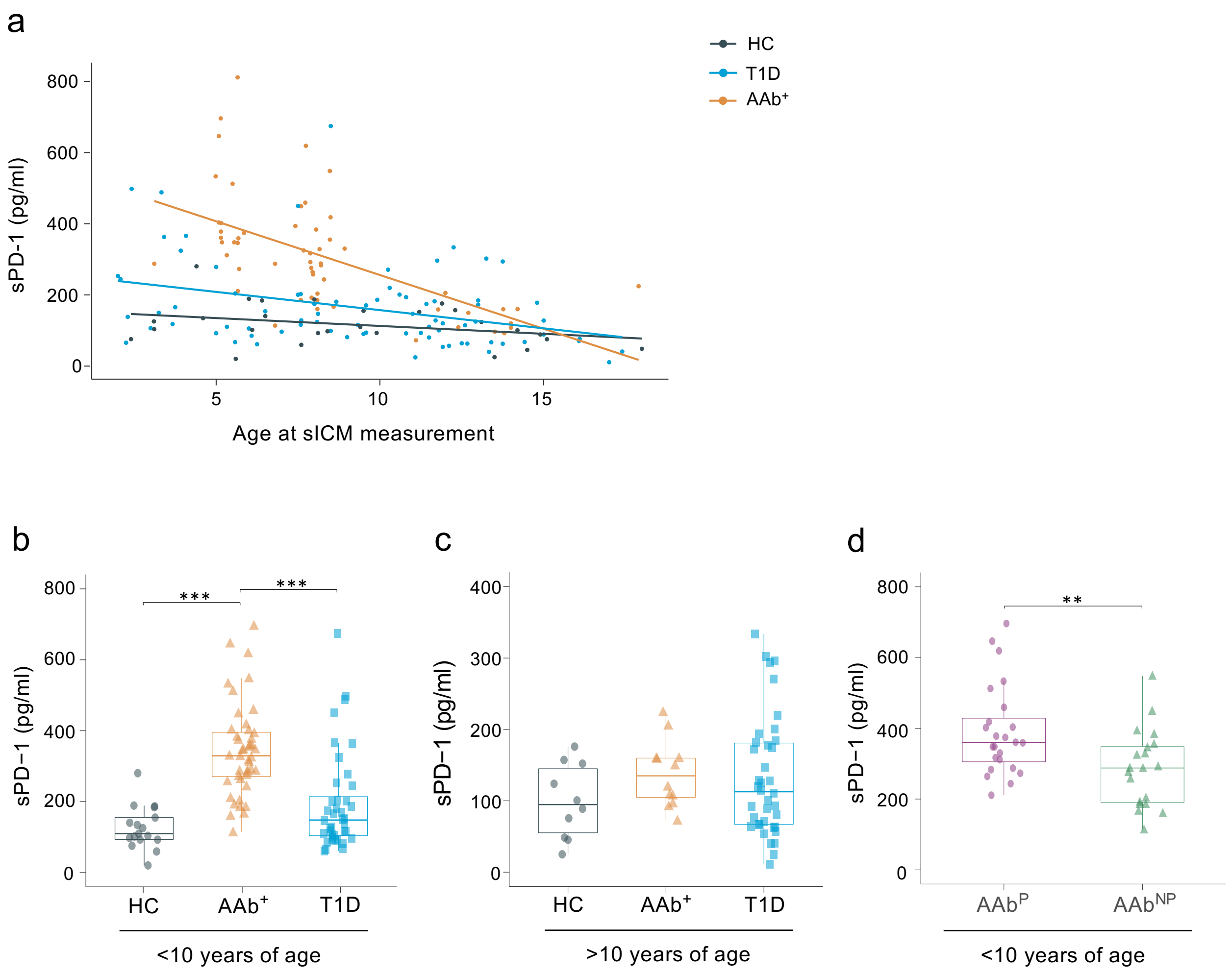

ESM Figure 1. sPD-1 concentrations negatively associated with age in AAb⁺ children, specifically in those under 10 years of age.

(**a**) Correlation between sPD1 concentrations (pg/ml) and age at time of sICM measurement in healthy children (HC, grey line and dots), islet-autoantibody positive (AAb⁺, orange line and dots) and type 1 diabetes (T1D, blue line and dots) children. R=-0.322 (*p*=0.102) for HC, R=-0.656 (*p*<0.001) for AAb⁺, R=-0.359 (*p*=0.001) for T1D by Pearson correlation. *p*<0.05 is considered statistically significant. (**b-c**) Box plots showing the distribution of sPD-1 concentrations (pg/ml) in healthy children (HC, grey circles), AAb⁺ (orange triangles) and type 1 diabetes (T1D, blue squares) under (**b**) or above (**c**) 10 years of age. Data are shown as the median (horizontal line in the box) and Q1 and Q3 (borders of the box). Whiskers show the lowest and highest values that are not outliers (i.e., data points below Q1 − 1.5 × IQR or above Q3 + 1.5 × IQR). Dots outside the whiskers represent outlier values. ****p*<0.001 by Mann-Whitney U test. (**d**) Box plots showing the distribution of sPD-1 levels in AAb⁺ children, under 10 years of age, who developed (AAb^P, violet circles) or did not develop (AAb^{NP}, green triangles) type 1 diabetes in the following years. Data are shown as the median (horizontal line in the box) and Q1 and Q3 (borders of the box). Whiskers show the lowest and highest values that are not outliers (i.e., data points below Q1 − 1.5 × IQR or above Q3 + 1.5 × IQR). Dots outside the whiskers represent outlier values. ***p*<0.01 by Mann-Whitney U test.
